# Effects of Shade and Leachate from Invasive *Chromolaena odorata* (Siam Weed) on Seedling Growth and Development of Native Tree Species in Nepal

**DOI:** 10.21315/tlsr2025.36.2.14

**Published:** 2025-07-31

**Authors:** Sunita Poudel, Ramesh Raj Pant, Mukesh Kumar Chettri, Lal Bahadur Thapa

**Affiliations:** 1 Central Department of Botany, Institute of Science and Technology, Tribhuvan University, Kathmandu, 44613 Nepal; 2 Central Department of Environmental Science, Institute of Science and Technology, Tribhuvan University, Kathmandu, 44613 Nepal; 3 Department of Botany, Amrit Campus, Tribhuvan University, Kathmandu, 44613 Nepal

**Keywords:** Siam Weed, Invasion, *Aegle marmelos*, Seedling Growth

## Abstract

The weed *Chromolaena odorata* has negative impacts on invaded ecosystems. Canopy of its aerial parts and allelochemicals released by the weed can suppress the growth and survival of native species. Field assessment of native trees *Aegle marmelos* and *Senegalia catechu* evidenced that a declining trend of their seedlings under higher canopy of *C. odorata*. Experiments carried out in pots revealed the negative effects of the weed’s leachate and shade on growth and development of *A. marmelos*. The leachate increased proline levels in *A. marmelos* seedlings by ca. 33% in shade and 43% in light. Specific leaf area and secondary roots were decreased significantly under both light and shade conditions by leachate. In the light condition, leachate reduced seedling biomass by ca. 26% and root length by 16%. Shade alone decreased overall seedling growth, including leaf area and biomass with poor root growth and increased specific leaf area. Results showed that *A. marmelos* is susceptible to shade and *C. odorata* leachate during its early stage of growth and development. In addition to other factors contributing to the decline of *A. marmelos* population in nature, the invasion of *C. odorata* intensifies the challenge. Our study clarifies that the invasion of *C. odorata* in native habitat has further contributed to the population decline of native species alongside other contributing factors in nature. Hence, there is an urgent necessity to control and manage *C. odorata* to protect native species. Removal of *C. odorata* from the invaded site will be beneficial for approaching light for native seedlings as well as preventing the leaching substances into the soil.

**Highlights**

- *Chromolaena odorata* (L.) R.M. King & H. Rob. is one of the highly problematic invasive alien species, which is responsible to harm native species.
- Shade and foliar leachate from invasive *C. odorat*a negatively impact the seedling growth and development of native tree *Aegle marmelos*.
- Removal of *C. odorata* from the invaded site is recommended to promote the recruitment and growth of seedlings of *A. marmelos*.

## INTRODUCTION

Alien plant invasion represents a serious environmental problem because of its multifaceted challenges, particularly its threat to native biodiversity and ecosystem functioning (Rai & Singh 2020; Gentili *et al.* 2021). The multifaceted effects include allelopathy of invasive species, competition for resources, loss of native diversity, disruption of ecological processes and alteration in soil quality in the habitats colonised by invasive species (Dogra *et al.* 2010). There are several key factors responsible to intensify the invasions, such as climate change, land use and land cover change, and other human activities like trades and transports, leading to significant challenges for the conservation of native biodiversity (Bradley *et al.* 2010; Bartz & Kowarik 2019). Among the world’s most threatening invaders, *Chromolaena odorata* (L.) R.M. King & H. Rob. (Siam weed), native to America, has been rapidly spreading across diverse habitats such as grasslands, forests, fallow lands and road sides worldwide (Zachariades *et al.* 2009).

The global spread of *C. odorata* has been significantly intensified with changing climatic scenarios (Adhikari *et al.* 2023). One of the important mechanisms of *C. odorata* to invade and compete with native species is allelopathy (Ambika & Jayachandra 1980). Studies have shown the allelopathic potential of *C. odorata* as it can release several harmful allelochemicals from aerial and underground parts that inhibit growth and development of neighbouring plants (Hu & Zhang 2013; Begum *et al.* 2021). Kato-Noguchi and Kato (2023) highlighted that *C. odorata* harms native plants’ seed germination and growth due to allelochemicals such as pyrrolizidine alkaloids, flavonoids, phenolic acids and terpenoids in the extracts, residues and exudations.

Besides allelopathy, *C. odorata* has been a successful invader also due to its high reproductive ability, rapid growth rate, adaptability to adverse environmental conditions and competitive ability with native species for resources (Ramakrishnan & Vitousek 1989; Koutika & Rainey 2010; Chandrasekaran & Swamy 2010). Additionally, the weed forms canopy in invading sites, creating shade over native herbs and seedlings, which may suppress growth and development of the herbs and growing seedlings of trees and shrubs (Thapa *et al.* 2016). Native plants under shade experience insufficient light, leading to elongation, chlorophyll deficiency and decreased photosynthesis (Veselkin *et al.* 2021; Honu & Dang 2000). Further, the stressed plants produce osmolytes such as proline as the sign of stressful environments. Such osmolytes play a crucial role in osmotic adjustment and they maintain cell turgor and water balance (Ábrahám *et al.* 2010).

In general, the interaction effect of both factors “canopy or shade and allelopathy” could be more severe than that caused by either one factor alone. However, responses vary among species as some species are shade-loving or shade-tolerant with different adapting mechanisms (Portsmuth & Niinemets 2007). The studies illustrating the effects of either one factor (shade) or interaction of both factors (shade and allelopathy) on native plants are limited on one side. On the other side, understanding species-specific responses to these factors is crucial for informed conservation strategies.

In Nepal, we observed a concerning situation, as mentioned above, where *C. odorata* had invaded a site of the tropical to sub-tropical regions vegetated by two important native trees, *Aegle marmelos* (L.) Corrêa and *Senegalia catechu* (L.f.) P. J. H. Hurter & Mabb. *Aegle marmelos* is a native tree having religious, food, medicinal and timber values (Pathirana *et al.* 2020). Its fruits are edible and as it has medicinal properties that enhance the immune system and cure various health conditions, including life-threatening diseases like cancer (Pathirana *et al.* 2020). Another species, *S. catechu* also has high economic value, which is used for extraction of dyes, wood and medicines (Jain *et al.* 2007). Both of these trees are common in the Manthali area of Ramechhap district of Bagmati Province, Nepal, where their habitat is heavily colonised by *C. odorata* (Poudel *et al.* 2024a).

These native trees are under increasing pressure of population decrease because of habitat loss, over-exploitation and changing environmental conditions (Plummer 2020; 2021). Based on field observation, we hypothesised that:

1. The toxic leachate of *C. odorata* inhibits the seedling growth and development of these native tree species.
2. The canopy of *C. odorata*, shades the forest floor and further suppresses the growth and development of the seedlings.

Then, we conducted experiments to confirm the mechanisms as hypothesised. Further, we aim to assess whether the native species demonstrates resilience or susceptibility to the shade and leachate effects of *C. odorata*. We selected *A. marmelos* as the model species for pot experiment because this species is categorised as a “Near Threatened Species” on the IUCN Red List due to its declining population (Plummer 2020).

## MATERIALS AND METHODS

### Study Site and Seedling Survey

**Figure 1:**
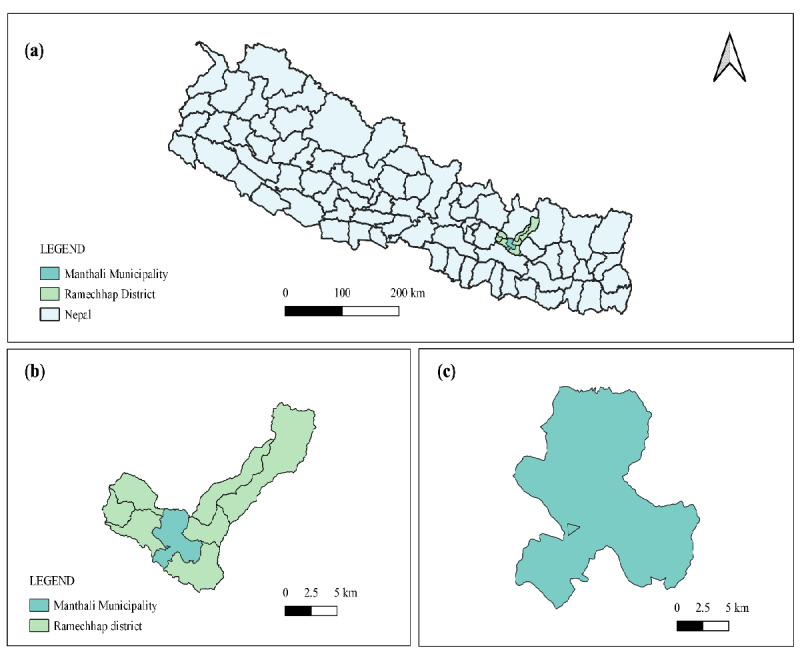
Map showing study site. (a) Map of Nepal, (b) Manthali Municipality and (c) Plots within Manthali Municipality.

The study comprises both a field survey and pot experiment. The field experiment assessed whether *C. odorata* has a negative impact on regenerating native seedlings. The survey was carried out during April 2022 in *C. odorata* invaded community forest, named Ramite Community Forest, which lies in 27°22′53.09″N to 27°23′3.65″N and 86°2′29.97″E to 86°2′27.28″E (elevation of 469–630 masl) at Manthali Municipality of Ramechhap district, Bagmati Province, Nepal (Fig. 1). The site has a typical upper tropical monsoon climate with an annual rainfall of 911.63 mm and an average temperature of 24.03°C (rainfall and temperature data of Manthali Station from 2014–2024, data provided by Department of Hydrology and Meteorology, Government of Nepal). The southern part of Ramechhap district lies in rain-shadow area of Mahabharat Hill, which experience drier conditions and is known as drought prone area (Shrestha *et al.* 2010).

The native trees *A. marmelos* (Rutaceae) and *S. catechu* (Fabaceae) are distributed in the study site (Photo plates I, II). For the seedling survey, square quadrats (size 5 m × 5 m each) were sampled along transects established within the forest, with each transect containing 5 to 6 plots positioned 200 m apart. The steep topography of the study site restricted transects and plot sampling to accessible sites. Within each plot, the *C. odorata* canopy cover was estimated visually, and the number of seedlings (≤ 1 m in height) was counted (Poudel *et al.* 2024a). The canopy covers of *C. odorata* were classified into two categories: (i) Outside canopy (canopy cover ranged from 0% to15%) and (ii) inside canopy (canopy cover > 15%) in each quadrat.

### Greenhouse Experiment

A greenhouse experiment was carried out in June 2022 with native *A. marmelos* to assess how shade, *C. odorata* leaf input (leachate), and their interactions affect the native seedlings. Seeds of *A. marmelos* were extracted from ripened fruits collected from a tree at 27°22′55.026+N, 86°2′14.412″E (elevation 521 masl) within the study forest. The seeds were air-dried, sealed in a zip bag, transported to the laboratory and stored in the refrigerator at 4°C until use. The seeds were scattered on moist filter paper and allowed to sprout in the dark at a temperature ranging from 25°C to 30°C with enough moisture. The seeds were germinated and seedlings reached up to 1.5 cm after one week of soaking.

Fresh leaves of *C. odorata* were collected from the Nepalthok area, Sindhuli district, Bagmati Province, Nepal (27.443°N and 85.818°E; 550 to 600 masl). The leaves were soaked in water (50 g of leaves/500 mL water) for 24 h. The leachate from the leaves mimicked the natural phenomenon of washing of the extract from the leaves (Poudel *et al.* 2024b).

Polyethylene pots of size 13 cm height and 11 cm diameter were prepared by filling 800 g of garden soil with a ratio of soil and sand 2:1 at Central Department of Botany, Tribhuvan University, Nepal. The seedlings of uniform size (1.5 cm long) were transplanted to the pots. Each of the pots contained one seedling. The seedlings were exposed to two major treatments:

1. Pots irrigated with normal water (tap water).
2. Pots irrigated with *C. odorata* foliar (leaf) leachate.

As the objective of the study was to know the effect of *C. odorata* canopy, we decided to simulate the canopy effect in the pot experiment by exposing plants to shade and full light conditions. The pots containing *A. marmelos* seedlings treated with tap water (control) and *C. odorata* leaf leachate were further exposed to two sub-treatments that were as follows:

#### Shade condition

A cardboard sheet having thickness of 6.5 mm was placed over the pots at the height of about 1 m to create shade for growing seedlings as the simulation of the canopy in the field. Light intensity in this condition was 50 to 60 lux.

#### Light condition

Seedlings were grown without shade with exposure to full light. The seedlings received light intensity ranging from 5,000 to 6,000 lux.

In this way, there were the following treatments:

1. Tap water + light (control).
2. Tap water + shade.
3. *C. odorata* leaf leachate + light.
4. *C. odorata* leaf leachate + shade.

The temperature of the greenhouse was 28°C–30°C and humidity ranged between 70%–85%. The pots were irrigated by water and leachate to respective pots (100 mL) in every alternate day after seedling transplantation. To reduce the positional effect, the pots were randomised every alternate day.

#### Measuring parameters

The plants were harvested after 60 days of transplantation. The number of leaves was counted, and the leaves were sampled for measuring the leaf area and specific leaf area (SLA). Mature leaves of each seedling were removed at the base of the petiole and their size (area) was measured using ImageJ software. Then, the leaves were oven-dried (80°C for 72 h) to obtain the dry mass. After that, SLA was measured using the formula: Leaf area/Dry mass (Vile *et al.* 2005).

Plants from the pots were removed gently without destroying the roots. The soil adhered around the roots was gently washed in tap water. The length of the shoot was measured from the base to the shoot apex, and the length of the primary root was measured from the point of origin to the root tip. The number of secondary roots arising from the primary root was counted for each seedling. Then, for the seedling biomass, both roots and shoots were dried in a hot air oven at 80°C for 24 h, and biomass was measured. In the leaves sampled, the proline was also analysed. The procedures of proline analysis followed the method of Bates *et al.* (1973).


$$\begin{array}{c} {}[(\mu \text{g proline}/\text{mL}\times \text{mL toluene})/115.5\\ \mu \text{g}/\mu \text{mole}]/[(\text{g sample})/5] \end{array}=\begin{array}{c} \mu \text{moles proline}/\text{g of fresh}\\ \text{weight material} \end{array}$$

### Statistical Analyses

Individual sample *t*-test was used to compare the seedling density of *A. marmelos* between inside and outside *C. odorata* canopies. Because of non-uniform data distribution, the Mann-Whitney U test was employed to compare the seedling density of *S. catechu* within and outside *C. odorata* canopies. Similarly, growth parameters of *A. marmelos* seedlings between (i) shade and light and (ii) water and leachate were also analysed using individual sample *t*-test. Poisson regression was used to compare the difference in the number of leaves and the numbers of secondary roots between the treatments. Principal Component Analysis (PCA) was used to evaluate the relationship of environmental parameters (shade/light and water/leachate) with the measuring parameters. All the statistical analyses were conducted in the software R (Version 4.3.1) (R Core Team 2023).

## RESULTS

### Effect of *C. odorata* Cover on Seedling Density of Native Trees

**Figure 2:**
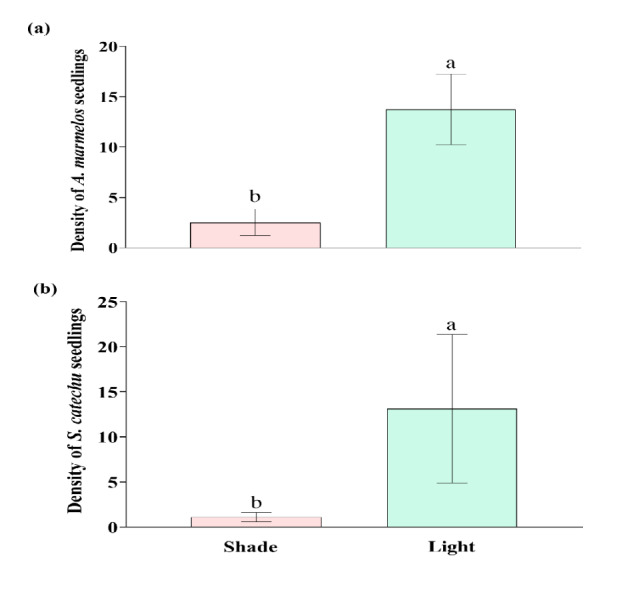
Density of (a) *A. marmelos* and (b) *S. catechu* seedlings per plot under the canopy *C. odorata* (Shade) and outside the canopy (Light). Different letters above the error bar indicate significant difference (*P* < 0.05).

The density of seedlings of both the native trees (*A. marmelos* and *S. catechu*) decreased significantly with increase in the canopy cover of *C. odorata* (*t* = −7.34, *P* < 0.001 for *A. marmelos* and *W* = 8, *P* = 0.012 for *S. catechu*) (Fig. 2). The density of the seedlings per plot was about six times lower inside the canopy of *C. odorata* compared of outside the canopy (Fig. 2). Comparing the densities between *A. marmelos* and *S. catechu*, the densities of both species were similar in outside of *C. odorata* canopy (i.e., 13.75 ± 1.24/plot for *A. marmelos* and 13.13 ± 8.25/plot for *S. catechu*). Interestingly, the density of *A. marmelos* was higher (2.5 ± 0.90/plot) than *S. catechu* (1.12 ± 0.99/plot) under *C. odorata* canopy.

### Effect of Leachate and Shade on Leaf Parameters of *A. marmelos*

**Figure 3:**
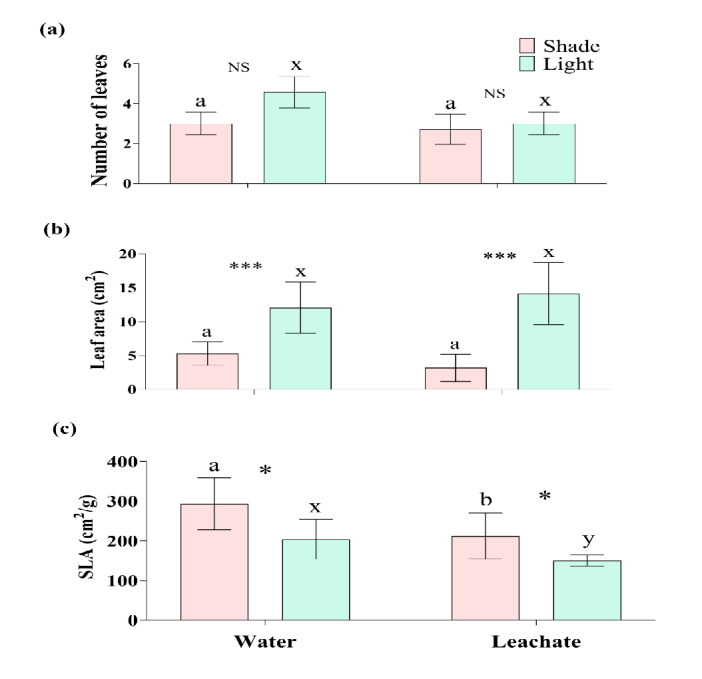
Effect of shade and leachate on leaf parameters: (a) Number of leaves, (b) Leaf area and (c) SLA of *A. marmelos* seedlings. Different letters above the error bar indicate significant differences between water and leaf leachate treatments (‘a–b’ for shade and ‘x–y’ for light). Significant differences between shade and light in each water and leachate treatment are denoted by * (*P* < 0.05) and *** (*P* < 0.001) and ‘NS’ represents non-significant differences between shade and light.

Number of leaves in *A. marmelos* did not vary between the seedlings grown in control and *C. odorata* leaf leachate under both shade and light conditions (*P* > 0.05) (Fig. 3a, Table 1). The leaf area was highly reduced under shade conditions when *A. marmelos* seedlings were grown with leachate (*t* = 5.78, *P* < 0.001) or without leachate (*t* = 4.31, *P* = 0.001) (Fig. 3b, Tables 2 and 3). The leaf leachate did not affect the leaf area of the seedlings in both light (*t* = 0.92, *P* = 0.375) and shade (*t* = 2.09, *P* = 0.059) conditions. On the contrary, SLA was decreased by leaf leachate (*t* = −2.71, *P* = 0.030 in light and *t* = −2.45, *P* = 0.030 in shade) while it was increased by shade (*t* = 2.86, *P* = 0.014 in water and *t* = −2.72, *P* = 0.031 in leachate) (Fig. 3c, Tables 2 and 3).

### Effect of Leachate and Shade on Shoot Length and Seedling Biomass

**Figure 4:**
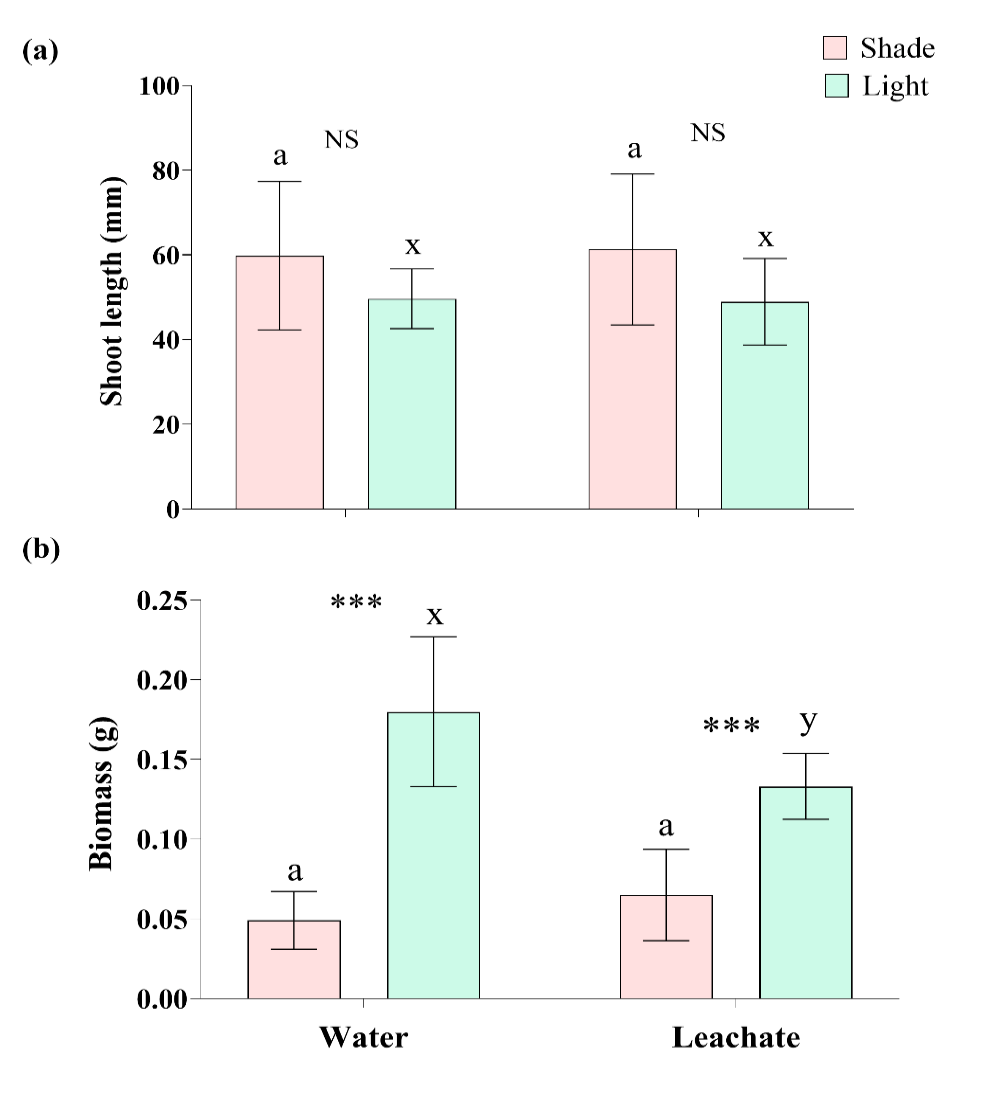
Effect of shade and leachate on: (a) shoot length and (b) seedling biomass of *A. marmelos* seedlings. Different letters above the error bar indicate significant differences between water and leaf leachate treatments (‘a–b’ for shade and ‘x–y’ for light). Significant differences between shade and light in each water and leachate treatment are denoted by *** (*P* < 0.001) and ‘NS’ represents non-significant difference between shade and light.

There was no change in shoot length due to the presence of *C. odorata* leachate (*t* = −0.15, *P* = 0.889 in light and *t* = 0.16, *P* = 0.876 in shade), and also, no change in shoot height was observed while comparing effects of shade and light with absence of the leachate (*t* = 1.42, *P* = 0.194) or presence of the leachate (*t* = 1.59, *P* = 0.137) (Fig. 4a, Tables 2 and 3).

The *C. odorata* leachate treatment in light significantly reduced the biomass of *A. marmelos* seedlings (*t* = −2.42, *P* = 0.032) but the leachate did not reduce the biomass under the shade (*t* = 1.24, *P* = 0.239) (Fig. 4b, Table 2). Comparing the biomass of seedlings between shade and light, the shade highly reduced the seedling biomass (*t* = −6.87, *P* < 0.001 in control treatment and *t* = −5.09, *P* < 0.001 in leachate treatment) (Fig. 4b, Table 3).

### Effect of Leachate and Shade on Root Length and Number

**Figure 5:**
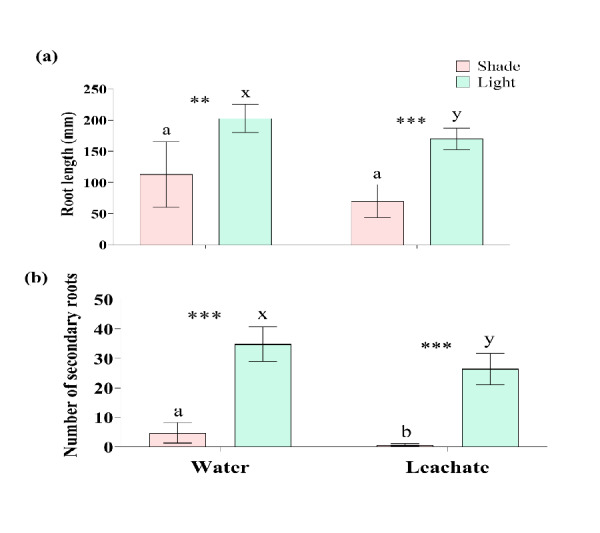
Effect of shade and leachate on: (a) root length and (b) number of secondary roots of *A. marmelos* seedlings. Different letters above the error bar indicate significant differences between water and leaf leachate treatments (‘a–b’ for shade and ‘x–y’ for light). Significant differences between shade and light in each water and leachate treatment are denoted by ** (*P* < 0.01) and *** (*P* < 0.001).

Contrary to shoot length, root length of *A. marmelos* seedlings were reduced by *C. odorata* leachate while they were exposed to full light (*t* = −3.08, *P* = 0.009), while the roots between water and leachate treatments were similar in length under shade (*t* = −1.94, *P* = 0.076) (Fig. 5a, Table 2). Roots of the seedlings under shade were shorter, comparing between shade and light conditions (*t* = −4.16, *P* < 0.001 in water and *t* = −8.37, *P* < 0.001 in leachate) (Fig. 5a, Table 3). Both the *C. odorata* leachate and shade were found to be harmful for the number of secondary roots in *A. marmelos.* The number of secondary roots was significantly decreased by shade and leaf leachate (*P* ≤ 0.001) (Fig. 5b, Table 1).

### Effect of Leachate and Shade on Proline

**Figure 6:**
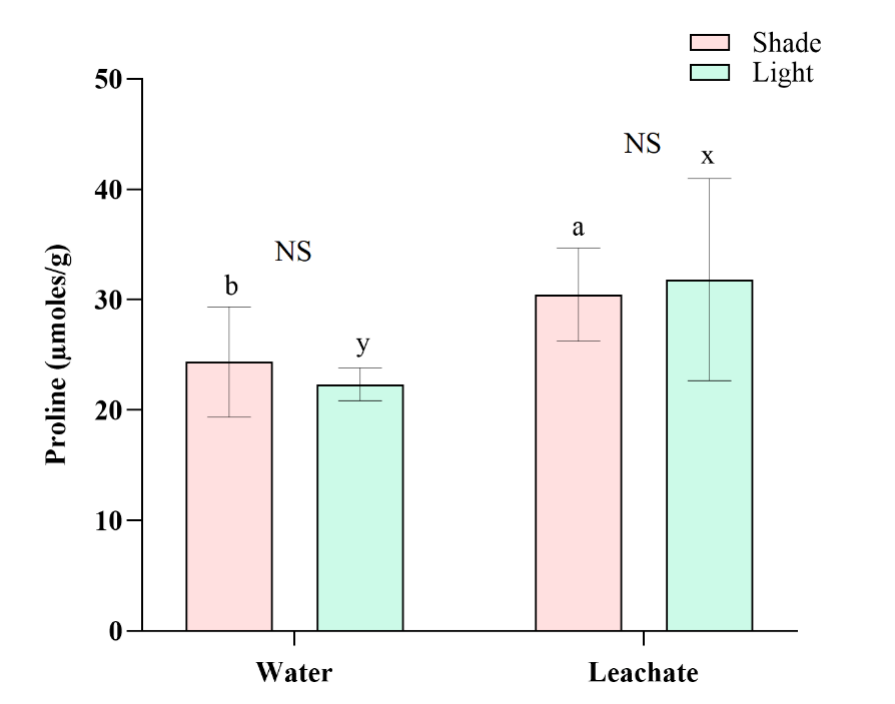
Effect of shade and leachate on proline concentration in *A. marmelos* seedlings. Different letters above the error bar indicate significant differences between water and leaf leachate treatments (‘a–b’ for shade and ‘x–y’ for light). ‘NS’ represents ‘non-significant’ results between shade and light in each water and leachate treatment.

Proline content was increased significantly in the seedlings grown with *C. odorata* leachate in both light (*t* = 2.71, *P* = 0.033) and shade (*t* = 2.49, *P* = 0.030) conditions (Fig. 6, Table 2). Variation in the concentrations of proline between shade and light was not significant (*t* = 1.03, *P* = 0.336 in water treatment and *t* = −0.36, *P* = 0.723 in leachate treatment) (Fig. 6, Table 3).

### Principal Component Analysis

**Figure 7:**
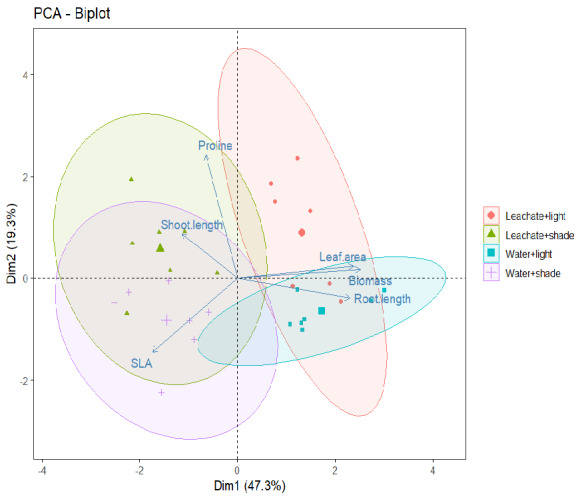
PCA biplot representing effect of shade and leachate of *C. odorata* on functional traits of *A. marmelos* seedlings.

PCA biplot displayed the first two principal components with 47.3% + 19.3% variations in growth parameters of *A. marmelos* seedlings by PC1 and PC2. The biplot indicates that *C. odorata* leaf leachate is associated with elevated proline levels, especially under shaded environmental conditions (Fig. 7). Although the leachate may still have some effect on proline under light conditions, the impact appears to be less pronounced than under shaded conditions. Regardless of whether seedlings received leachate or water, shoot length and SLA were affected by shade. Similarly, irrespective of water or leachate treatment, light exposure promotes longer root length, more leaf area and higher total biomass production (Fig. 7).

## DISCUSSION

This study compares the seedling recruitment status of two highly valuable and threatened native species between *C. odorata* invaded and uninvaded sites. The study evaluates potential mechanisms of interference of *C. odorata* on the selected seedling growth and development. The seedlings of native *A. marmelos* and *S. catechu* were found to be declined in their habitat invaded by *C. odorata.* When the seedlings of *A. marmelos* exposed to leachate of *C. odorata* with shade environment, the plants were found to be stressed as indicated by elevated level of proline concentrations and impacted seedling growth parameters negatively.

The higher density of seedlings of both *A. marmelos* and *S. catechu* outside the canopy of *C. odorata* compared to inside the canopy (Fig. 2) suggests a significant negative impact of this invasive weed on seedling establishment of these native trees in nature. Regression analysis carried out by Poudel *et al.* (2024a) had shown a declining trend of seedling number of *A. marmelos* on increasing *C. odorata* cover. The seedling density between two canopy classes of *C. odorata* (< 15% and higher) also revealed similar results. We further compared density of *A. marmelos* with *S. catechu* to understand how two different native species respond to *C. odorata*. Result showed that the densities of both species are almost similar in open canopies, but the density of *S. catechu* were about 55% lower than *A. marmelos* under *C. odorata* high canopies. It indicated that *S. catechu* seedlings might have been more impacted by *C. odorata*, comparing to *A. marmelos*.

One of the commonly known reasons for the negative impact could be allelopathy. *Chromolaena odorata* releases allelochemicals that inhibit the seed germination and seedling growth of other plants, suppressing seedling establishment (Ambika & Poornima 2004; Kato-Noguchi & Kato 2023). Further, a dense stand of *C. odorata* is seen in the invaded sites that reduce the light availability, limiting the resources, which are crucial for seedling growth and development. The result clearly indicates that *C. odorata* is responsible for declining seedling recruitment of the threatened species in nature.

In the nature, the allelochemicals can be extracted by rainwater and added to soil as leachate from *C*. *odorata* aerial parts, while aerial parts also create shade over growing native seedlings. The experiment designed in this study mimicked the natural environment. As expected, the study illustrated the impacts of *C. odorata* leachate, shade, and light on *A. marmelos* seedlings.

Although *C. odorata* leachate used in this study did not show a statistically significant effect on the number of leaves of *A. marmelos*, the result indicates that the number can be decreased under higher concentrations of the leachate, more prominent in the light conditions (Fig. 3a). Also, the higher concentrations of leachate may reduce *A. marmelos* leaf size in shade and contrastingly, the size can be increased in light (Fig. 3b).

Interestingly, the leachate was responsible for decreasing the SLA in both light and shade conditions; the leaves exhibited an increase in SLA when subjected to shade (Fig. 3c). This trend of impacts implies that the allelopathic activities and response of the recipient plant depend on light intensity (Nakai *et al.* 2014). Plants change their SLA in response to intensities of light and show a tendency to increase their SLA when shaded to intercept more light (Liu *et al.* 2016; Buajan *et al.* 2017). Results of our study suggest that the response of *A. marmelos* seedlings to *C. odorata* leachate is also modulated by light intensity. The SLA of *A. marmelos* was markedly reduced by *C. odorata* leachate under dry conditions comparing to control (Poudel *et al.* 2024a).

Dense stands of *C. odorata* create shade and under the shade, fewer and smaller leaves of *A. marmelos* with low SLA are expected, according to our results. Conversely, if the seedlings of *A. marmelos* grow in an open canopy and receive leachate of *C. odorata*, their leaves are expected to be fewer but larger in size with low SLA (Fig. 3c). Despite the attempts by the seedlings of *A. marmelos* to increase their SLA against reduced leaf number and size, the cumulative effect of shade and *C. odorata* leachate appears to have a negative impact. This unfavourable outcome is likely to add the challenge of seedling recruitment for *A. marmelos.* It suggests that the seedlings’ ability to adapt in stressful environment is limited due to potentially hindering overall growth and survival. Consequently, the ability of *A. marmelos* to recruit seedlings in high canopy areas may be significantly impaired, which may further affect population dynamics.

Many plant species exhibit the traits of shade avoidance under the canopy by elongating their seedlings to penetrate the canopy (Fenner 1978). *A. marmelos* seedlings elongate in shade compared to light, but the difference was not significant, and also, shoot length was not affected by *C. odorata* leachate (Fig. 4a). This result signifies that both factors have no influence on shoot elongation during early growth of *A. marmelos*. Interestingly, the longer seedlings grown in the shade have significantly low biomass compared to the seedlings grown in the light. Simultaneously, the leachate led to reduce the seedling biomass in light conditions (Fig. 4b). This result also suggests the critical role of shade and light in influencing the toxicity of leachates on plant biomass accumulation. As discussed in the leaf parameters, the seedlings with longer shoot but low biomass (low reserve food) in shade may encounter difficulties in survival and establishment because the seedlings with greater biomass survive better (Tsakaldimi *et al.* 2013). Similar projections can be made for the seedlings influenced by *C. odorata* leachate and grown in open canopy (light) (Figs. 4 and 7). Under canopy, seedlings may be more susceptible to the toxic effects of leachates and conversely, in light conditions, plants may be better able to cope with the stress. This highlights the importance of light intensity in mitigating leachate toxicity.

Root architecture and morphology are greatly influenced by light variations (Miotto *et al.* 2021). Both the longer roots and large number of secondary roots can enhance uptake of nutrients and water from the soil, help to stabilise the plants and improve drought tolerance (Wang *et al.* 2006; Zia *et al.* 2021). Our study showed that the root length in *A. marmelos* declined in shade and the length be likely to decrease with the addition of *C. odorata* leachate (Fig. 5a). Similarly, the formation of secondary roots was severely inhibited by both the factors (shade and leachate) (Fig. 5b). These suggest that the seedlings of *A. marmelos* face significant stress during establishment and growth under shade conditions, regardless of the presence of *C. odorata* leachate. The results indicated that the seedlings of *A. marmelos* in the *C. odorata* invaded sites have been facing a detrimental impact of shade and toxic leachate on root development.

### Leachate Elevates Proline Levels in *A. marmelos* but not by Shade

Increasing concentration of proline in plants is mainly influenced by increased glutamate levels, which is a known stress-mitigating response (Verbruggen & Hermans 2008; Hayat *et al.* 2012). This study aimed to know whether the seedlings of *A. marmelos* perceive the shade and *C. odorata* leachate as the stressors, stimulating the seedlings to cope by releasing proline. As expected, the seedlings responded to the leachates by releasing a significantly high concentration of proline in both light and shade environments (Fig. 6). The leachate of *C. odorata* might have increased osmotic stress in seedlings rather than the shade and therefore, the seedlings had increased proline as an osmoprotectant to maintain water balance (Agduma & Sese 2016). Zhang *et al.* (2018) highlighted that the leachate from invasive plants can damage cellular membranes and alter the osmotic potential of cells.

Chen *et al.* (2015) found that the growth of tobacco seedlings was inhibited by the allelochemical (juglone) released from walnut tree and in response; the plants increased proline concentration. They have highlighted that the proline can lessen juglone stress in tobacco. Our study also showed that the seedlings of *A. marmelos*, when exposed to *C. odorata* leachate, likely attempted to mitigate allelochemical stress by secreting proline. The toxicity of the leachate was prominent on leaves, plant biomass, and roots of *A. marmelos* seedlings. The seedlings likely responded to the leachate as a primary stressor and it is expected that the toxicity would be severe in the absence of proline. However, other undetected factors may also influence secretion of proline in the seedlings, despite our efforts to minimise such variables.

Overall, the seedlings of native trees are under stressful conditions in the *C. odorata* invaded areas. The pot experiments ensured that the observed trends were consistent under controlled conditions. This study further confirmed the negative effects of *C. odorata* leaf leachate on *A. marmelos* seedlings growth and proline production as shown by Poudel *et al.* (2024a). The evaluation of shade impact by this study on the seedlings of *A. marmelos* has given additional insight with foliar leachate of *C. odorata*. Given our findings of a decline in seedling density of *S. catechu* by *C. odorata* invasions, specifically those greater than *A. marmelos*, further studies can help to better understand the underlying causes. As both of the trees are high valuable as explained in introduction, immediate action to control *C. odorata* should be taken to conserve these native plants. Delaying the action will further endanger vulnerable plant species in nature. Physical removal at the first stage, and then integrated approaches are effective options for controlling *C. odorata* (Goodall & Erasmus 1996). Frequent removal of *C. odorata* facilitates the growth of seedlings of native plants, enabling them to ultimately form canopy that suppresses further growth of *C. odorata*.

## CONCLUSION

In conclusion, *Chromolaena odorata* invasion has increased the challenge to the seedling recruitment of threatened native trees. Seedlings of *A. marmelos* demonstrate enhanced performance in the presence of light and absence of *C. odorata* leachate. Examining the impact of shade and leachates from the invasive plant on the early growth of native trees provides a valuable insight into how the invasive species influence the recruitment of threatened tree seedlings. Results suggest that *A. marmelos* is susceptible to shade and allelopathic effects of *C. odorata*. The invasion of *C. odorata* in the native habitat has further contributed to the population decline of *A. marmelos* alongside other contributing factors in nature. Therefore, light exposure for the growth and development of native seedlings by removing *C. odorata* is recommended. This approach also prevents the leaching of substances from the weed into the soil. Such insights are valuable for managers to initiate conservation activities to mitigate the threats to native plant populations and to restore native habitats.

## Figures and Tables

**Table 1:** Poisson regression statistics for number of leaves and roots of *A. marmelos.*

Between water and leachate				

	Shade		Light	

	*z*-value	*P*-value	*z*-value	*P*-value
Number of leaves	0.32	0.752	0.79	0.432
Number of secondary roots	3.93	**< 0.001**	2.84	**0.004**

Between light and shade				

	**Water**		Leachate	

	*z*-value	*P*-value	*z*-value	*P*-value

Number of leaves	−1.50	0.134	−1.04	0.299
Number of secondary roots	−10.79	**< 0.001**	−7.97	**< 0.001**

Note*: P*-values in bold indicate statistical significance.

**Table 2:** *t*-test statistics for seedling growth parameters of *A. marmelos* between treatments of water and *C. odorata* leachate in light and shade.

Functional traits	*t*-value	df	*P*-value

	Light		
Leaf area	0.92	12	0.375
Specific leaf area	−2.71	6.96	**0.030**
Shoot length	−0.15	12	0.889
Seedling biomass	−2.42	12	**0.032**
Root length	−3.08	12	**0.009**
Proline	2.71	6.32	**0.033**

	Shade		

Leaf area	2.09	12	0.059
Specific leaf area	−2.45	12	**0.030**
Shoot length	0.16	12	0.876
Seedling biomass	1.24	12	0.239
Root length	−1.94	12	0.076
Proline	2.49	12	**0.030**

*Note: P*-values in bold indicate statistical significance.

**Table 3:** *t*-test statistics for seedling growth parameters of *A. marmelos* between light and shade conditions

Functional traits	*t*-value	df	*P*-value

	Water		
Leaf area	4.31	12	**0.001**
Specific leaf area	2.86	12	**0.014**
Shoot length	1.42	7.90	0.194
Seedling biomass	−6.87	7.74	**< 0.001**
Root length	−4.16	12	**< 0.001**
Proline	1.03	7.06	0.336

	Leachate		

Leaf area	5.78	12	**< 0.001**
Specific leaf area	−2.72	6.72	**0.031**
Shoot length	1.59	12	0.137
Seedling biomass	−5.09	12	**< 0.001**
Root length	−8.37	12	**< 0.001**
Proline	−0.36	12	0.723

*Note: P*-values in bold indicate statistical significance
